# 

**Published:** 1852-05

**Authors:** 


### CONTENTS.

### ORIGINAL COMMUNICATIONS.

After-Pains, Observations on, . . . . . . 151
Asiatic Cholera, . . . . . . 298, 348
Alcohol a Remedy for Bite of Rattlesnake, . . . . 389
Blood, Effects of Impaired Condition of, . . . . . 103

Cancerous Disease, Nature and Cure of, . . . . 1
Cancrum Oris, Case of, . . . . . . . . . 11
Chloroform, applied locally in Fractures, . . . . . 13
Chronic Diarrhœa of California, . . . . . . 61
Chloride of Sodium in Visceral Obstructions, . . . . 388
Cured by the Kick of a Horse, . . . . . . . 391
Colica Pictonum, . . . . . . . . . 492

Dysentery, . . . . . . . . 14, 385
Diagnosis by Physical Signs, Uncertainty of, . . . . 56
Dysentery, on the Treatment of, . . . . . . 108
Dysentery, Report on, . . . . . . . . 292

Epidemic Dysentery, . . . . . . . . 493

Fevers in Marquette Co., Wis., . . . . . . 198
Fracture of Lower Jaw, treated successfully, . . . . 204
Fever Produced by Decomposition of Potatoes, . . . . 433
Fever and Dysentery, Observations on, . . . . . 247

Hypertrophy and Disease of Kidney, . . . . . 9
Hæmorrhagic Diathesis, Singular Effects of, . . . . 110
Hydrothorax, . . . . . . . . . 145
Hereditary Cataracts, Operation for Relief of, . . . . 355

Intestinal Fistulæ, Report of Two Cases of, . . . . 535

Knee Joint, Removal of Foreign Body from, . . . . 147

Lightning, a Case of Injury by, . . . . . . . 289

Morning Sickness in Pregnancy, . . . . . 12
Monstrosity, Cause of, . . . . . . . 111

Opium, . . . . . . . . . . . . 71

Popular Prejudices in the Treatment of Measles, . . . . 204
Prolonged Exercise of the Vocal Organs as a means of Strengthening them, . . . . . . . . . . . 241

Rupture of the Intestine, without External Laceration, . . . 259
Rapid Growth and Spontaneous Expulsion of Hydatids, . . 496
Report of three cases, with unusual symptoms, . . . . 539

Spontaneous Hæmorrhage, Pathology of, . . . . 49, 97
Strangulated Hernia, Tobacco Smoke in, . . . . . 78
Scirrhus Lung, Case of, . . . . . . . 205
Specific Nature of Erysipelas, and its Connection with Puerperal Fever, 194
Spina Bifida, Case of . . . . . . . . . 109
Strangulated Hernia not attended by the Usual Symptoms, . 154
Spontaneous Evolution. Case of, . . . . . . 202

Tape-Worm, New Article for Treatment of, . . . 203
Treatment of Milk-Sickness, . . . . . . . 13
The Liver and its Secretions, . . . . . . 63
Typhoid Fever, . . . . . . . . . . 337

Uterine Hæmorrhage, consequent upon want of Contractile Power in the Uterus, . . . . . . . . . 152
Uterine Hæmorrhage, . . . . . . . . 497

Vaginal Speculum, Benefits from the Use of, . . . . 257
Valedictory Address, . . . . . . . . 481

### EDITORIAL.

Apology, . . . . . . . . . . 32
American Medical Association, Meeting of, . . . . . 43
Æsculapian Society at Palestine, Semi-Annual Meeting of, . . 92
Amputation of Lower Jaw, . . . . . . . 182
American Medical Association, Transactions of, . . . . 515
Acchromatic Microscopes, . . . . . . . 95
American Medical Association, . . . . . . . 335

Bird on Urinary Deposites, . . . . . . . 34
Beck's Lectures on Materia Medica and Therapeutics, . . . 81
Bartlett on Fevers, . . . . . . . . 230
Books Received, . . . . . . . . . 240, 475
Braithwaite's Retrospect, . . . . . . . . 475

Common Salt in Scarlet Fever, . . . . . . 46
Canada Medical Journal, . . . . . . . . 47
Cook County Medical Society, . . . . 87, 137, 231, 526
Cook County Medical Society, Proceedings for April, . . . 565
Cook County Medical Society, . . . . . . . . 568
Contributions to Experimental Physiology, . . . . 177
Clinical Reports on Continued Fever, . . . . . 225
Com. of Am. Med. Assoc. on the Epidemics of Illinois, . . . 239
Chronic Disease of the Heart, Treatment of, . . . . 285, 325
Clinical Lectures U. S. Marine Hospital, . . 225, 328, 377, 429, 477
Cooper's Lectures on the Principles and Practice of Surgery, . 375
Carpenter's Principles of Human Physiology, . . . . 428

Discourse on the Times, Character, and Writings of Hippocrates, . 422
Dental Medicine, a Practical Treatise on, . . . . . 525

Empirical Remedies, Essay on, . . . . . . 182
East Tennesee Record of Medicine and Surgery, . . . 85
Exsection and Disarticulation of Lower Jaw, Claims to Priority, . 182

Formula, . . . . . . . . . . 46

Flint on the Variations of Pitch in Auscultation and Percussion, . 470

Graham's Elements of Chemistry, . . . . 229
Hancock County Medical Association, . . . . . 334
Hæmorrhagic Diathesis, . . . . . . 38

Illinois State Medical Society, . . . . , 33, 90
Iowa University Again, . . . . . . . 184
Intermittent and Remittent Fever, . . . . . 235
Items and News, . . . . . . . . . 348
Ink for the Million, . . . . . . . . 336

Knox County Medical Society, . . . . . 143

List of Graduates in Rush Medical College, . . . . 527

Medical Schools, . . . . . . . 239
Muguet, . . . . . . . . 236
Medical Meetings, . . . . . 232
Meigs' Velpeau's Midwifery, . . , . . . 130
Materia Medica for Students, Review of, . . . . 86
Medical Student's Vade Mecum, . . . . . . 84
Medical Statistics, . . . . . . . . 47
Medical News, . . . . . . . 192
McHenry County Medical Society, . . . . . 332
Medical Department of Michigan University, . . . 335
Miller's Principles and Practice of Surgery, . . . 375

Ninth Annual Report of the Managers of the Lunatic Asylum of the State of New York, . . . . . . . 32
New Orleans Medical Register, . . . . . . . 48
Notice of Wood's Practice of Medicine, . . . . . 129
Neligan on the Diseases of the Skin, . . . . . 473
New Medical Journals, , . . . . . . 476

Observations made in the United States Marine Hospital, . 234
Obituary, . . . . . . . . . 96, 348
Obituary, . . . . . . . 568

Pereira's Materia Medica and Therapeutics, . . 230
Pneumonia, . . . . . . . . . 134
Practical Chemistry, a Branch of Medical Education, . . 182
Physician's Visiting List, . . . . . . . 324

Rees' Analysis of Physiology, . . . . . . 86

Report on the Easton Lunatic Asylum, Va., . . . . 130
Ranking's Half-Yearly Abstract, . . . . . . 260, 475
Rush Medical College, . . . . . . . 335

St. Louis University, Address to the Graduates of, . . 182
Simon's General Pathology, . . . . . . 427

Typhoid Fever at Almont, Mich., . . . . . . 474
Tape Worm, . . . . . . . . . . 238
To the Profession, . . . . . . . . 31
Tartaric Acid used for Aiding the Solution of Di-Sulph. Quin., . 96
The Liver and its Diseases, . . . . . . . 40
Transactions of the Illinois State Medical Society, for 1852, . 272
Third annual announcement of the University of Michigan, . 187
To our Readers, . . . . . . . . 240
Tully's Materia Medica, . . . . . . . 475

Transactions of the Medical Society of the State of Missouri, . 321
Transactions of the Medical Society of the State of Pennsylvania, . 324
The Ash, a Remedy in Rheumatism and Gout, . . . 383

University of Iowa and the American Medical Association, . . 94
Urine, Agent for Detecting the Quantity of Urea in the, . 287

Varix, New Treatment of, . . . . . . . . 240
Whiteside County Medical Society, . . . . 191
Works Received, . . . . . . . . . 48
What Foreigners think of us, . . . . . . . 131
Wilson on Syphilis, . . . . . . . . 417

SELECTIONS.

Albuminous Urine, . . . . . . . . . 26
Asthenic Dropsy, Treatment of. . . . . . 28
American Medical Association, Proceedings of, . . . . 65
A Memoir on the Pathology and Treatment of Leucorrhœa, . 211
Ague Treated by Terebinthinate Liniments, . . . . 273
Alcohol, a Remedy for the Poison of the Rattlesnake, . . . 317
Aneurism of the Aorta, Curative Treatment of, . . . . 318
Accidents Caused by Chloroform, Method of Remedying, . . 367
Arsenic, a Case of Poisoning by Two Ounces, . . . . 393
Artificial Abortion to Save the Mother, . . . . . 406
Arterial Hœmorrhage Averted by Flexion of the Limbs, . 175

Blistering in Gleet, . . . . . . . . . 73
Bronchitis, . . . . . . . . . . 165
Belladonna a Remedy in Pertussis, . . . . . . 305
Bowels, Remedy for Intussusception of, . . . . . 313
Blood, Changes in Disease, . . . . . . 315
Bernard's Theory of Hepatico-Renal Circulation, Review of, . 408
Bone, Nerves of the, . . . . . . . . 415
Binocular Microscope, . . . . . . . . 462
Bleeding in Pneumonia, Rules for, . . . . . . 30
Bite of Rattlesnake, . . . . . . . . 168
Belladonna a Palliative in Epilepsy, . . . . . . 128
Bite of a Rattlesnake, . . . . . . . . 550

Chloroform in Delirium Tremens, . . . . . . 20
Calomel and Soda as a Cathartic, . . . . . 77
Collodion in Erysipelas, . . . . . . . . 128
Cod-Liver Oil in Phthisis, . . . . . . . 172
Chloroform, Treatment for Hemicrania and Neuralgia, . . 274
Chloroform in Obstruction of the Bowels from Spasm, . . . 368
Catarrhal Pneumonia and Lobar Pneumonia of Children, . . 407
Chemist's Dream, . . . . . . . . . . 452
Chronic Diarrhœa, Persesquinitrate of Iron in, . . . . 371
Chemical Disinfectants, . . . . . . . . 465, 499
Cancer, Treated by Lactate of Iron, . . . . . 157
Cod Liver Oil, New way of taking, . . . . . . 274

Democracy and Doctors, . . . . . . . . 161
Dysentery, Treatment of, . . . . . . . . 271
Death of Dr. Drake, . . . . . . . . 400
Deafness, Glycerine in the Treatment of, . . . . . 171
Dysentery, Turpentine in, . . . . . . 80

Drunkenness, Saline Injections for the Cure of, . . . 80
Dysentery, Iodine Clysters in the Treatment of, . . 127

Extraordinary Fecundity, . . . . . . . . 274
Excision of Uvula, Snoring Prevented by, . . . 416
Epidemic of Puerperal Fever, Prophylaxis of, . . . 76

Fellis Bovini, as a Medicine, . . . . . . . 268
Ferro-Phosphas-Calcis in Pulmonary Consumption, . . . 396
Function of Spleen and Lymphatic Glands as Secretors of Blood, 410
False Aneurism of Femoral Artery, . . . . . 459

Hœmatology, New Researches on, . . . . . . 551
Hats and Baldness, . . . . . . . . 553
Hooping-Cough and Asthma, Nitric Acid in, , . . . 125

Iodine Injections in the Peritoneum in Ascites, . . . 28
Itch Cured in two hours, . . . . . . . 30
Inhalation of Chloroform, Treatment of Pneumonia by, . . 126
Infantile Paralysis, . . . . . . . . 371
Intussusception of the Bowels, Remedy for . . . . 313

Iodide of Potassium, as a Remedy for the Affections caused by Lead and Mercury, . . . . . . . . . . 546

Lungs, On Spinal Acid of . . . . . . , 224
Liver, On the Structum, Functions and diseases of, . 207
Lancing Gums, Death from, . . . . . . 174
Lactation Reproduced, by application of child to breast, . . 22

Menses, Sinapisms to Mammæ for Suppression of, . . 73
Musca Volantes, on the Cause and Diagnostic value of, . . 222
Maintenance of Life in limbs separated from the body, . . 261
Manganese an Adjuvant to Iron, . . . . . . . 265
Medicinal Substances on the Tongue instead of into the Stomach, 395
Molecular Origin of the Tissues, . . . . . . 413
Microscopic Observations respecting Arterial and Capillary circulation, . . . . . . . . 120

New Remedies, Observations on the Use of, . . . 357

Obstinate Ulcers, Treatment of, . . . . . . . 463
Oxalic Diathesis, . . . . . . . 502
Oxygenated Bitters, , . . . . . . . 502
Opium, . . . , . . . . . 557

Post Mortem Examination of the late Daniel Webster, . . 505
Professional Aphorisms, . . . . . . . . 414
Phthisis, on the Hereditary Transmission of, . . . . 320
Phthisis, Cod Liver Oil in, . . . . . . . 172
Peurperal Convulsions, Cold Water in, . . . . . 25
Painful Tubucle, . . . . . . . . . . 221
Pneumonia, new Symptom of, . . . . . . . 223
Phthisis, the influence of Pregnancy upon, . . . . . 316
Persusquintrate of Iron in Chronic Diarrhæ, . . . . . 311
Professional Letters from Paris, . . . , . . . 362
Pregnant Woman, influence of Imagination on the . . . 123
Psoriasis Pulmaris, Cured by Iodide of Arsenic, . . . 397
Physiology of the Liver, . . . . . . . . . 402
Prophylactic effects of Quinine against Erysipelas, . , 216

Quinine in Typhus and Typhoid Fevers, . . , 79

Resection of Superior Maxillary and Malar Bones, . . . 160
Rapping Mania, . . . . . . . 555

Sesqui Carbonate of Ammonia in Lapra and Psoriasis, . . 127
Spinal Cord, Reparative power of, . . . . . . 117
Subcutaneous Nævus cured by Vaccination, . . . 24
Substitutes for Mercurials in the material of Syphillis, . . 29
Still-born Children, new mode of Treating, . . . 415
Sartrate of Soda as a Purgative, . . . . . 562

Traumatic Spasms, . . . . . . . 412
Types in Fevers, Blending and Conversion of, . . . 449
The Medical and Physical Sciences, . . . . . . 542

Urine, Death from a dose of, . . . . . . 176
Urethra, Strictures of, . . . . . . . 369

Veratrum Veride, an Arterial Sedative, . . . 319
Vaccination, on the Duration and Protecting power of, . . 373
Valuable Substitute for Ergot, . . . . . . 561
Vibratile Cells in the Bronchial Mucous Membrane, . . 416

Wound of the Internal Iliac Artery, caused by instruments in labor, 457
Wry Neck cured without Cutting, . . . . . 511
Writing Prescriptions, . . . . . . . 503

*Works received.*

The Annual Discourse before the Phil. Co. Med. Society, by Samuel Jackson, M.D.

Nelson's Northern Lancet.

The Western Journal of Med. and Surg.

The New Orleans Monthly Med. Register, edited by A. Forster Axson, M.D.

The Western Lancet and Hospital Reporter.

The New Jersey Medical Reporter.

The Medical Examiner and Record of Med. Science.

The Medical News and Library.

The St. Louis Med. and Surgical Journal.

The Nashville Journal of Medicine and Surgery.

The Stethoscope and Vir. Medical Gazette.

The Transylvania Medical Journal.

The Charleston Med. Journal and Review.

The Boston Medical and Surgical Journal.

The Canada Medical Journal.

The East Tennessee Record of Medicine and Surgery.

The Am. Journal of Med. Sciences.

### RECEIPTS

### FOR THE JOURNAL DURING THE MONTHS OF MARCH & APRIL.

### Illinois.

| | | |
|---|---|---|
| Dr. | J. Higgins, | $2 00 |
| " | H. Trusedale, | 1 00 |
| " | G. S. Barrows, | 2 00 |
| " | D. D. Thompson, | 1 00 |
| " | W. S. Knight, | 1 00 |
| " | F. R. Payne, | 2 00 |
| " | B. S. Robinson, | 4 00 |
| " | A. Comstock, | 2 00 |
| " | W. D. Nelson. | 2 00 |
| " | A. W. Heize, | 2 00 |
| " | I. J. Haslett, | 1 00 |
| " | R. A. Rosenberger, | 1 00 |
| " | T. G. Klepper, | 2 00 |
| " | W. K. Palmer, | 1 00 |
| " | W. H. Davis, | 2 00 |
| " | J. A. Tyler, | 2 00 |
| " | W. R. Griswold, | 2 00 |
| " | L. W. Warren, | 2 00 |
| " | W. T. Linn, | 2 00 |
| " | R. R. Stone, | 2 00 |
| " | E. Woodruff, | 2 00 |
| " | S. R. Mason. | 2 00 |
| " | C. H. Ritchings, | 2 00 |
| " | M. Sparks, | 2 00 |
| " | E. S. Cooper, | 5 00 |
| " | H. K. Freeman, | 1 00 |
| " | E. C. Ellitt, | 2 00 |
| " | F. Blades, | 2 00 |
| " | J. I. Bullock, | 2 00 |
| " | J. Bowen, | 2 00 |
| " | I. T. Anthony, | 2 00 |
| " | O. S. Jenks, | 2 00 |
| " | Guy Hulitt, | 2 00 |
| " | F. Scammon, | 2 00 |
| " | H. Martin, | 2 00 |
| " | M. Brown, | 4 00 |
| " | J. Brinkerhoff, | 4 00 |
| " | A. Eldridge, | 4 00 |
| " | S. A. Paddock, | 2 00 |
| " | W. W. Welch, | 1 00 |
| " | Z. H. Madden, | 1 00 |
| " | P. Green, | 3 00 |
| " | L. Fuller, | 1 00 |
| " | W. W. Barnes, | 1 00 |
| " | F. T. Miner | 5 00 |
| " | D. Proctor | 2 00 |
| " | W. F. Coleman, | 4 00 |
| " | E. K. Farmer, | 4 00 |
| " | J. T. Bissell, | 2 00 |
| " | C. M. Daniels, | 5 00 |
| " | S. G. Marrs, | 2 00 |
| " | R. Rouse, | 2 00 |
| " | D. W. Boyd, | 2 00 |
| " | A. A. Smith, | 2 00 |
| " | R. Mills, | 2 00 |
| " | F. B. Haller, | 2 00 |
| " | W. D. Craig, | 2 00 |
| " | J. W. Phelps, | 6 00 |
| " | A. Patterson, | 5 00 |

### Ohio.

| | | |
|---|---|---|
| Dr. | T. Ballard, | $4 00 |
| " | L. Q Ranson, | 4 00 |
| " | O. White | 4 00 |

### Indiana.

| | | |
|---|---|---|
| Dr. | J. M. Justice, | $6 00 |
| " | G. J. Bentley, | 7 00 |
| " | W. W. Crowder, | 2 00 |
| " | W. W. Taylor, | 7 00 |
| " | J. S. Collins, | 2 00 |
| " | S. M. Linton, | 2 00 |
| " | D. Hartman, | 1 00 |
| " | G. W. Reddick, | 4 00 |
| " | ——Townsend | 2 00 |
| " | W. F. Boone, | 2 00 |
| " | A. Carley, | 4 00 |
| " | S. Loften, | 4 00 |
| " | Geo Bolles, | 4 00 |
| " | W. R. Godfrey, | 1 00 |
| " | J. M. Stucky | 4 00 |
| " | R. D. Mansy, | 2 00 |
| " | A. M. Lewis, | 2 00 |
| " | R. C. Pierce, | 4 00 |
| " | M. C. Wood, | 2 00 |
| " | A. B. Buchanan, | 2 00 |
| " | S. C. Hoyne, | 2 00 |
| " | W. T. Green, | 2 00 |
| " | B. Bartholomew, | 2 00 |
| " | C. H. Butler, | 4 00 |
| " | C. Brachett, | 2 00 |
| " | D. A. D. Sargent, | 3 00 |
| " | J. E. Moler, | 4 00 |
| " | E. Jones, | 7 00 |
| " | L. D. Hogle, | 2 00 |
| " | J. Hicks, | 2 00 |
| " | B. F. Kieth, | 2 00 |
| " | C. H. Davis, | 2 00 |
| " | F. W. Tibbs, | 4 00 |
| " | W. Brenton, | 2 00 |

### Wisconsin.

| | | |
|---|---|---|
| Dr. | E. Rider, | $2 00 |
| " | T. R. Kibbe, | 4 00 |
| " | E. G. Little, | 2 00 |
| " | H. Warne, | 2 00 |
| " | H. S. Beeson, | 2 00 |
| " | C. S. Millington, | 2 00 |
| " | Gaylor & Beals, | 1 00 |
| " | S. S. Clark, | 2 00 |
| " | J. Parker, | 2 00 |
| " | W. P. M'Alister, | 2 00 |
| " | A. H. Vannorstrand, | 2 00 |
| " | D. A. Colton, | 2 00 |
| " | E. Hopkins, | 2 00 |

### Michigan.

| | | |
|---|---|---|
| Dr. | J. P. Hall. | $2 00 |
| " | J. G. Cornell, | 2 00 |
| " | H. Davis, | 2 00 |
| " | A. F. Stevens, | 3 00 |
| " | R. B. Porter, | 4 00 |
| " | J. W. Hall. | 2 00 |
| " | J. K. Finley, | 2 00 |
| " | Cornell, | 8 00 |
| " | N. Miller, | 2 00 |

### N Carolina.

| | | |
|---|---|---|
| Dr. | J. H. Saunders, | $4 00 |

### Iowa.

| | | |
|---|---|---|
| Dr. | J. Acres, | $2 00 |
| " | Hall, | 2 00 |
| Dr. | Rinehart, | 4 00 |
| " | F. Brady, | 1 00 |
| " | Miller & Noies, | 4 00 |

### PROSPECTUS

### OF THE NEW SERIES OF THE

### NORTH-WESTERN

### MEDICAL AND SURGICAL JOURNAL.

EDITED BY

**W. B. HERRICK, M.D.,**

*Professor of Anatomy and Physiology in Rush Medical College; Surgeon to the U.S. Marine Hospital; and one of the Surgeons of the Ill. General Hospital.*

ASSISTED BY

**H. A. JOHNSON, M.D.**

THE NORTH-WESTERN MEDICAL AND SURGICAL JOURNAL will be published hereafter, on the first of each month, beginning in May, in numbers, each containing, exclusive of advertisements, Forty-eight pages, closely printed, with new type ordered expressly for this work; thus presenting at the end of the year a volume of nearly Six Hundred pages.

The change from a bi-monthly to a monthly Journal, has been made in accordance with the expressed wishes of numerous subscribers.

The editor, with his associate, Dr. JOHNSON, who will devote his time exclusively to the Journal, will endeavor, in addition to a full and complete Domestic Summary, to present numerous extracts from English, and translations from French and German Medical periodicals.

In addition to the Communications from Contributors, whose favors, it is hoped, will be continued, the Original Department will contain, from time to time, reports of Cases treated, and of Observations made, in the two Hospitals, now fully organized in Chicago.

### TERMS:

**TWO DOLLARS PER ANNUM, IN ADVANCE.**

As an inducement to Physicians and others to act as Agents, in extending the circulation of the Journal,

| | | | |
|---|---|---|---|
| Three copies will be furnished to new subscribers for | | | $5 00 |
| Five | " | " | 8 00 |
| Seven | " | " | 10 00 |

### TERMS OF ADVERTISING.

| | |
|---|---|
| One page, One year ............$20,00 | One page, single insertion........$5,00 |
| One-halt page, One year, ...... 12,00 | One-half page, single insertion.... 3,00 |

☞ *Letters must be post-paid, and directed to the* "NORTH-WESTERN MEDICAL AND SURGICAL JOURNAL, *Chicago, Ill.*" *A list of receipts will appear in each No.*

